# Liquid chromatography-mass spectrometry-based metabolomic profiling reveals sex differences of lipid metabolism among the elderly from Southwest China

**DOI:** 10.1186/s12877-023-03897-z

**Published:** 2023-03-21

**Authors:** Yuan-Jun Huang, Wei Ke, Ling Hu, You-Dong Wei, Mei-Xue Dong

**Affiliations:** 1 The First Branch, The First Affiliated Hospital of Chongqing Medical University, Chongqing, 430060 China; 2 Department of Neurology, Renmin Hospital of Wuhan University, Hubei General Hospital, No.238 Jiefang Road, Wuchang District, Wuhan, Hubei China; 3 Department of Neurology, The First Affiliated Hospital of Chongqing Medical University, No.1 Youyi Road, Yuzhong District, Chongqing, 400016 China

**Keywords:** Metabolomic analysis, Sex difference, The elderly, Lipid metabolism, Metabolite sets enrichment analysis

## Abstract

**Background:**

The sexual dimorphism represents one of the triggers of the metabolic disparities while the identification of sex-specific metabolites in the elderly has not been achieved.

**Methods:**

A group of aged healthy population from Southwest China were recruited and clinical characteristics were collected. Fasting plasma samples were obtained and untargeted liquid chromatography-mass spectrometry-based metabolomic analyses were performed. Differentially expressed metabolites between males and females were identified from the metabolomic analysis and metabolite sets enrichment analysis was employed.

**Results:**

Sixteen males and fifteen females were finally enrolled. According to clinical characteristics, no significant differences can be found except for smoking history. There were thirty-six differentially expressed metabolites between different sexes, most of which were lipids and lipid-like molecules. Twenty-three metabolites of males were increased while thirteen were decreased compared with females. The top four classes of metabolites were fatty acids and conjugates (30.6%), glycerophosphocholines (22.2%), sphingomyelins (11.1%), and flavonoids (8.3%). Fatty acids and conjugates, glycerophosphocholines, and sphingomyelins were significantly enriched in metabolite sets enrichment analysis.

**Conclusions:**

Significant lipid metabolic differences were found between males and females among the elderly. Fatty acids and conjugates, glycerophosphocholines, and sphingomyelins may partly account for sex differences and can be potential treatment targets for sex-specific diseases.

## Background

Sexual dimorphism is a common biological phenomenon in the nature. Robust differences are found between males and females in disease incidence, disease severity, metabolism, and pharmacodynamics of interventions. The prevalence rates of coronary heart disease, heart failure, stroke, and various metabolic syndromes are significantly higher among the males while the occurrence and severity of knee osteoarthritis are also influenced by sex, with older females affected to a greater degree by the disease compared to age-matched males [1]. Sex difference is also found in lipid and cholesterol metabolism. At homeostasis, the female is prone to incorporate free fatty acids into triglycerides whereas the male likely oxidizes circulating free fatty acids [2]. It is reported that sexual dimorphism is due to sex chromosome and the following sex-specific hormone action.

Recognition and identification of sex differences are important for researchers to develop new treatments and physicians to deal with sex-specific diseases. Metabolomic is a systematic analysis of all the metabolites in a biological sample. The metabolites identified by metabolomic include amino acids, peptides, oligonucleotides, carbohydrates, organic acids, ketones, aldehydes, lipids, steroids, alkaloids, xenobiotics, and any other small molecules deriving from biological processes. The plasma metabolome of healthy individuals has already been analyzed and it is reported that sex differences are mainly correlated with amino acids and acylcarnitines, including creatine [3]. Sex can also affect the metabolome of biological fluids in an age-dependent way and previous publications identified the interaction between sex and age [4]. However, little publications emphasized aged population, as metabolism changes following the sex-specific hormones in postmenopausal females. Furthermore, the metabolic fingerprint of sex differences also varies between different races [5]. The published researches are mainly from western countries including Caucasian, African-American, Hispanic, and so on [6].

Herein, we adopted untargeted liquid chromatography-mass spectrometry (LC–MS)-based metabolomics to analyze sex-specific metabolic changes in a Chinese aged population. Plasma is chosen in the metabolomic analysis as it is a relatively accessible, stable, and informative biofluid.

## Methods

### Participants

A group of aged healthy population were recruited in Department of Physical Examination, the First Affiliated Hospital of Chongqing Medical University, from April 2016 to February 2017. All the participants were more than 50 years old and all the included females should be postmenopausal. The participants were without any acute illness or in acute state of chronic diseases at the enrollment.

This study was approved by the ethics committee of the First Affiliated Hospital of Chongqing Medical University and performed in accordance with Declaration of Helsinki. Statements of informed consent were obtained from all the participants prior to inclusion in this study. Clinical characteristics and metabolomic analysis were blindly collected or performed, separately [7].

### Clinical characteristics

Clinical characteristics of all the participants were collected, including age, smoking history, alcohol consumption, hypertension, diabetes mellitus, hypercholesterolemia, and coronary heart disease. Fasting plasma samples were obtained by puncture of the median cubital vein at 6:00 am. The levels of total cholesterol, triglyceride, high-density lipoprotein cholesterol, low-density lipoprotein cholesterol, apolipoprotein A1, apolipoprotein B, and lipoprotein a were also determined using a Cobas Integra 400 plus automatic biochemical analyzer with matched reagent kits (Roche, Basel, Switzerland) [8].

### Metabolomic analysis

The detailed procedure of metabolomic analysis was described in the former research [9]. Firstly, plasma samples stored at -80℃ were gradually thawed on ice, 2-chloro-1-phenylalanine dissolved in methanol (0.3 mg/mL) was served as internal standard. In a 1.5 mL Eppendorf tube, 50μL sample and 10μL internal standard were added and then vortexed for 10 s. Subsequently, 150μL ice-cold mixture of methanol and acetonitrile (2/1, vol/vol) were added. The mixtures were vortexed for 1 min, ultrasonicated at ambient temperature (25℃) for 5 min, placed at -20℃ for 10 min, and centrifuged at 15000 rpm at 4℃ for 10 min. 100μL of the supernatants from each tube were collected, filtered through 0.22 μm microfilters, and transferred to LC vials. The vials were stored at -80℃ until LC–MS analysis. Quality control sample was obtained by mixing all the samples equally as a pooled sample, and then processed using the above method with the analytic reagents. Fifteen quality controls and the whole samples were randomly injected throughout the analytical run to provide a set of data from which repeatability can be assessed.

We adopted a Waters UPLC I-class system equipped with a binary solvent delivery manager and a sample manager, coupled with a Waters VION IMS Q-TOF Mass Spectrometer equipped with an electrospray interface (Waters Corporation, Milford, USA) to perform the untargeted LC–MS metabolomics. Acquity BEH C18 column (100 mm × 2.1 mm i.d., 1.7 μm; Waters Corporation) was used and the column temperature was maintained at 45℃. The separation process was achieved using the following gradient: 5% B—25% B over 1–1.5 min, 25% B -100% B over 1.5–10.0 min, 100% B – 100% B over 10.0 – 13.0 min; 100% B – 5% B over 13.0 – 13.5 min, and 13.5 – 14.5 min holding at 5% B at a flow rate of 0.4 mL/min, where B is acetonitrile (0.1% (vol/vol) formic acid) and A is aqueous formic acid (0.1% (vol/vol) formic acid). Injection volume was 3μL.

The mass spectrometric data was collected using the Waters mass spectrometer operating in either positive or negative ion mode. The source temperature and desolvation temperature was set at 120℃ and 500℃, respectively, with a desolvation gas flow of 900 L/h. Centroid data was collected from 50 to 1,000 m/z with a scan time of 0.1 s and interscan delay of 0.02 s over a 13 min analysis time. Centroid data was collected from 50 to 1,000 m/z with a scan time of 0.1 s and interscan delay of 0.02 s over a 13 min analysis time. The obtained data were processed by baseline filtering, peak identification, integration, retention time correction, peak alignment and normalization using the build-in metabolomic software Progenesis QI (Waters Corporation). Retention time ranged from 0.5 to 14.0 min, mass ranged from 50 to 1000 Da, and mass tolerance was 0.01 Da. Isotopic peaks were excluded for analysis, noise elimination level was set at 10.00, minimum intensity was set to 15% of base peak intensity, and retention time tolerance was set at 0.01 min.

After that, data sets including m/z, peak retention time, and peak intensity of each ion were obtained, and further reduced by removing any peaks with missing values in more than 60% of the total samples. The internal standard was used for data quality control. Metabolite identification was performed based on the following two steps. First, we used our self-constructed metabolite databank, which contains chemical standards and a manually curated compound list based on accurate mass (m/z, ± 5 ppm), retention time, and spectral patterns. Second, further metabolites were identified based on accurate mass, isotope pattern and MS/MS spectra against public databanks, including Metlin (https://metlin.scripps.edu), Human Metabolome Database (HMDB, http://www.hmdb.ca) and so on. The peak intensity was deemed as expression level of a metabolite [10].

The positive and negative peak data were merged and multivariate statistical analyses were performed by the SIMCA-P 13.0 software package (Umetrics, Umea, Sweden). The quality control samples were used to validate the stability of the metabolomic analysis. The unsupervised principal component analysis (PCA) was used to observe the data distribution. The orthogonal partial least squares-discriminant analysis (OPLS-DA) model with sevenfold cross validation was constructed to show statistical differences and recognize differentially expressed metabolites between the two groups. The constructed model was validated by a response permutation test with 200 iterations. Metabolites with variable influence on projection values (obtained from the OPLS-DA model) of greater than 1.0, fold change values of greater than 1.5 or lower than 0.67, and *p* values (obtained from Student *t* test) of less than 0.05 were recognized to be differentially expressed. Partial least squares-discriminant analysis (PLS-DA) with sevenfold cross validation was further constructed based on the above differentially expressed metabolites to visualize their differential ability between the male and females. The differentially expressed metabolites and their quantities were then exhibited as clustering heatmap. Metabolite sets enrichment analysis was further performed based on the above metabolites using MetaboAnalyst 5.0 (metaboanalyst.ca) [11].

### Statistical analysis

Statistical analyses were completed using a commercially available software package (IBM SPSS version 22.0, New York, USA). Continuous data were expressed as means ± standard deviation and compared using Student *t* tests. Categorical data were exhibited as absolute numbers and percentage (%), and analyzed using Pearson χ^2^-tests or Fisher exact tests. *P* values less than 0.05 were considered as statistical significances [12].

## Results

### Clinical characteristics

A total of sixteen males and fifteen females were finally included in this study. No significant difference can be found in the mean age of these participants (62.56 ± 2.43 versus 65.73 ± 1.96). The males had a significant higher rate of smoking history compared with the females. There were no significant differences in the other clinical characteristics, indicating the two groups of participants were comparable. No statistical significances were found in total cholesterol, triglyceride, high-density lipoprotein cholesterol, low-density lipoprotein cholesterol, apolipoprotein A1, apolipoprotein B, and lipoprotein a (Table 1).

**Table 1:** Clinical characteristics of all participants with different sexes included in this study

Variable (SEM/%)	Male (16)	Female (15)	t value	*p* Value
Age (year)	62.56 ± 2.43	65.73 ± 1.96	-1.009	0.312
Smoking (%)	9 (56.3%)	0	11.889	0.001
Alcohol consumption (%)	4 (25%)	0	2.368	0.124
Hypertension (%)	8 (50%)	4 (26.7%)	1.777	0.183
Diabetes mellitus (%)	1 (6.3%)	1 (6.7%)	0.000	1.000
Hypercholesterolemia (%)	7 (43.8%)	2 (13.3%)	2.157	0.142
CHD (%)	1 (6.3%)	2 (13.3%))	0.003	0.953
TC (mmol/L)	4.516 ± 0.196	4.333 ± 0.115	0.790	0.174
TG (mmol/L)	1.739 ± 0.111	1.411 ± 0.138	1.860	0.477
HDL-c (mmol/L)	1.200 ± 0.088	1.318 ± 0.085	-0.962	0.812
LDL-c (mmol/L)	2.984 ± 0.203	2.826 ± 0.119	0.657	0.097
Apo-A1 (g/L)	1.297 ± 0.055	1.366 ± 0.064	-0.820	0.738
Apo-B (g/L)	0.985 ± 0.069	0.873 ± 0.034	1.423	0.054
Lpa (mg/L)	181.33 ± 62.97	186.57 ± 75.35	-0.054	0.920

*SEM* standard error of the mean, *CHD* coronary heart disease, *TC* total cholesterol, *TG* triglyceride, *HDL-c* high-density lipoprotein cholesterol, *LDL-c* low-density lipoprotein cholesterol, *Apo-A1* apolipoprotein A1, *Apo-B* apolipoprotein, *Lpa* lipoprotein a

### Metabolomic analysis

**Fig. 1:**
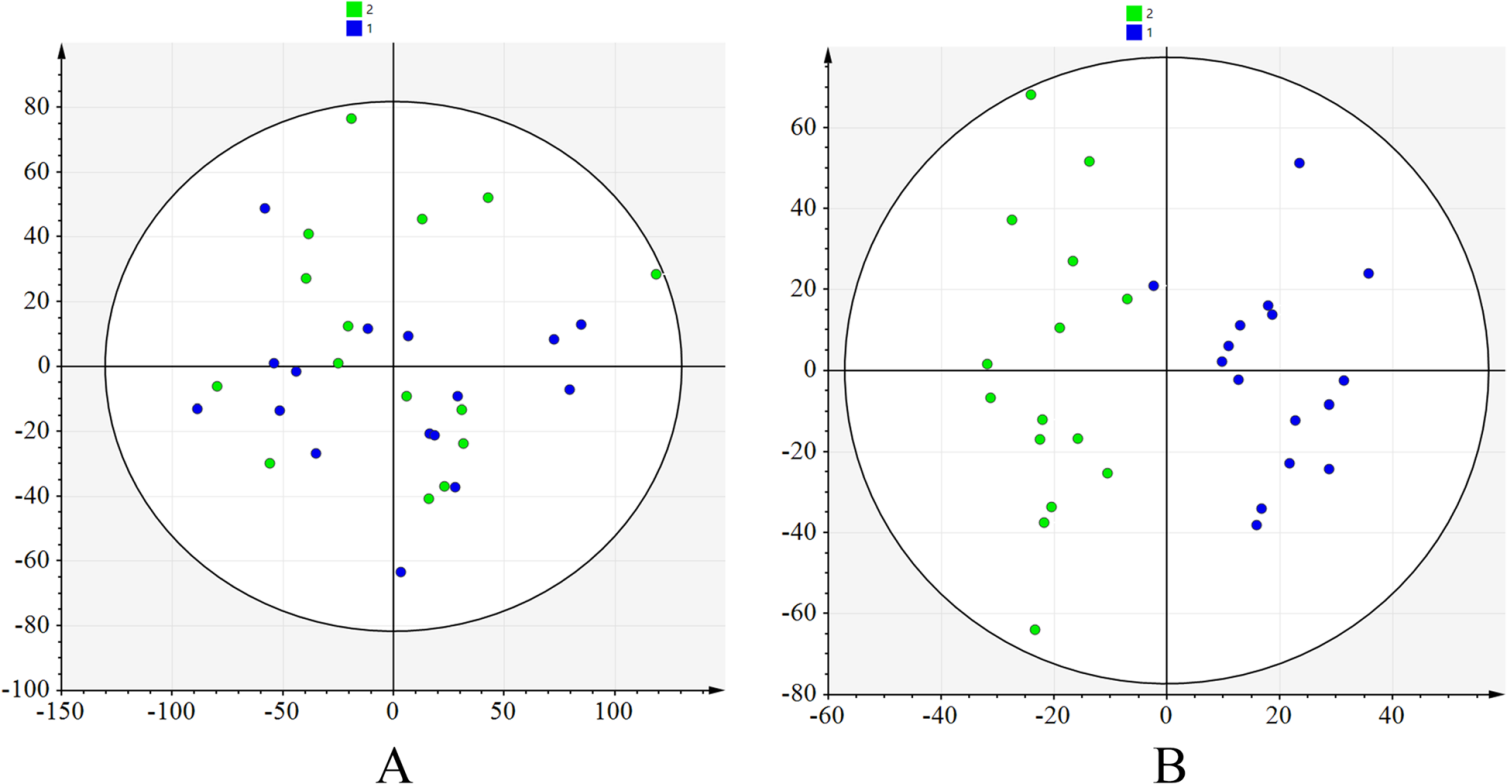
Multivariate statistical analyses of the liquid chromatography-mass spectrometry-based metabolomics between males and females included in this study. **A** PCA score plot of all the participants included seven components with a R^2^X value of 0.661 and a Q^2^ value of 0.219. **B** OPLS-DA score plot of all the participants included two components with a R^2^X value of 0.129, R^2^Y value of 0.855, and Q^2^ value of 0.084. PCA, principal component analysis; OPLS-DA, orthogonal partial least squares-discriminant analysis

After excluding internal standards, a total of 10,403 individual peaks, including 6040 positive and 4363 negative peaks, were detected in approximately 98.8% of samples in each group. Based on these peaks, score plots from PCA and OPLS-DA analysis were performed and the results showed separations between the two groups (R^2^X = 0.129, R^2^Y = 0.855, Q^2^ = 0.084) (Fig. 1).

**Fig. 2:**
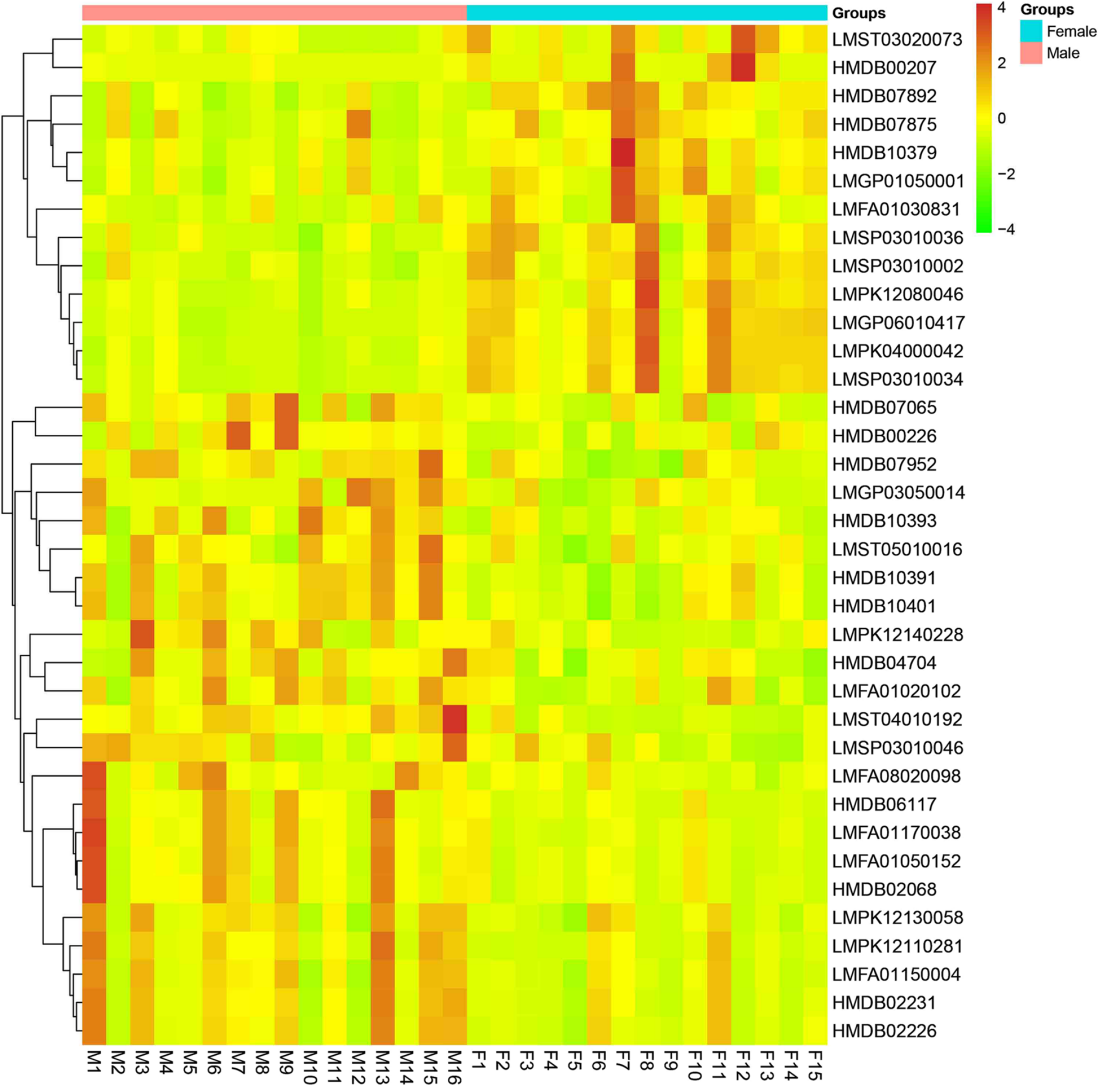
Clustering heatmap of differentially expressed metabolites between males and females

**Fig. 3:**
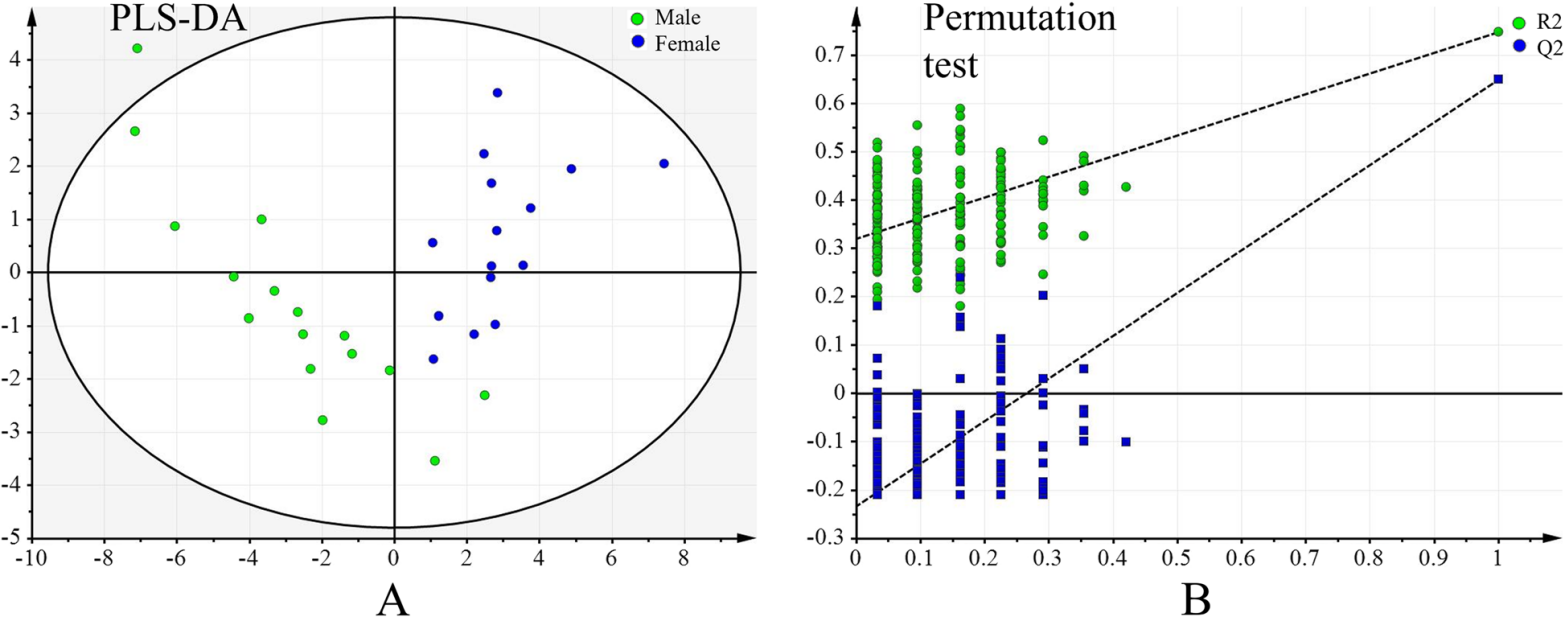
PLS-DA score plot and its corresponding response permutation test based on the differentially expressed metabolites between males and females in this study. **A** PLS-DA score plot of all the participants included two components with a R^2^X value of 0.516, R^2^Y value of 0.748, and Q^2^ value of 0.65, indicating the model was stable and reliable. **B** Response permutation test indicated the constructed PLS-DA model was not over-fitting (R^2^ = (0.0, 0.311), Q^2^ = (0.0, -0.237)). PLS-DA, partial least squares-discriminant analysis

There were thirty-six differentially expressed metabolites between the two sexes, most of which were lipids and lipid-like molecules. Twenty-three metabolites were increased in the males while thirteen were decreased (Table 2). The differentially expressed metabolites and the corresponding quantities of each sample were exhibited in clustering heatmap (Fig. 2). PLS-DA score plot indicated clear separations between two sexes based on the above differentially expressed metabolites. The score plot included two components with a R^2^X value of 0.516, R^2^Y value of 0.748, and Q^2^ value of 0.65, indicating the model was stable and reliable. Meanwhile, the corresponding response permutation test indicated the PLS-DA model was not over-fitting (R^2^ = (0.0, 0.311), Q^2^ = (0.0, -0.237)) (Fig. 3).

**Table 2:** Differentially expressed metabolites between males and females based on the untargeted liquid chromatography-mass spectrometry-based metabolomic analysis

Class	Compound ID	Common name	m/z	RT (min)	MS	Ion mode	VIP	FC	*p* value
Fatty Acids and Conjugates	LMFA01150004	3-carboxy-4-methyl-5-propyl-2-furanpropanoic acid	503.190	7.870	36	Positive	2.167	0.516	0.026
	HMDB02231	Eicosenoic acid	309.280	7.865	38.7	Negative	2.151	0.522	0.027
	HMDB02226	Adrenic acid	377.269	7.865	36.7	Negative	2.157	0.534	0.027
	HMDB02068	Erucic acid	337.312	8.303	37.7	Negative	2.308	0.410	0.018
	LMFA01020102	2-methyl-2E-heptenoic acid	283.191	4.343	38.7	Negative	2.079	0.576	0.034
	LMFA01170038	Tricosanedioic acid	405.300	8.303	37.8	Negative	2.323	0.318	0.018
	HMDB04704	9,10-DHOME	313.239	5.583	37.3	Negative	2.053	0.664	0.035
	LMFA01050152	methyl 4-[2-(2-formyl-vinyl)-3-hydroxy-5-oxo-cyclopentyl]-butanoate	531.220	8.301	38.4	Positive	2.243	0.333	0.022
	LMFA08020098	N-palmitoyl tyrosine	837.600	9.570	36.3	Negative	2.143	0.520	0.029
	LMFA01030831	26:5(11Z,14Z,17Z,20Z,23Z)	431.318	5.830	35.7	Negative	2.323	2.401	0.021
	HMDB00207	Oleic acid	327.254	6.789	37.5	Negative	2.071	8.631	0.050
Glycerophosphocholines	HMDB10391	LysoPC(20:1(11Z))	572.370	6.321	38.5	Positive	2.857	0.626	0.002
	HMDB10401	LysoPC(22:4(7Z,10Z,13Z,16Z))	616.363	6.316	58.5	Negative	2.888	0.640	0.002
	HMDB10393	LysoPC(20:3(5Z,8Z,11Z))	590.347	6.248	37.9	Negative	2.312	0.578	0.017
	HMDB07952	PC(15:0/22:1(13Z))	822.599	9.284	40.4	Negative	2.819	0.670	0.003
	HMDB07875	PC(14:0/18:3(6Z,9Z,12Z))	772.514	8.317	33.3	Negative	2.184	1.695	0.025
	LMGP01050001	PC(13:0/0:0)	452.279	5.151	56.9	Negative	2.562	1.619	0.010
	HMDB10379	LysoPC(14:0)	468.308	5.149	37.3	Positive	2.640	1.556	0.009
	HMDB07892	PC(14:0/22:6(4Z,7Z,10Z,13Z,16Z,19Z))	800.520	8.357	44.3	Positive	3.413	1.719	0.000
Sphingomyelins	LMSP03010046	SM(d18:0/17:0)	717.592	9.407	37.2	Negative	1.930	0.568	0.049
	LMSP03010034	SM(d18:2/14:0)	695.508	8.191	57.6	Positive	3.916	1.526	0.000
	LMSP03010002	SM(d18:1/12:0)	691.505	8.159	57.2	Negative	3.413	1.680	0.000
	LMSP03010036	SM(d18:2/15:0)	731.535	8.385	52.1	Negative	2.951	1.704	0.003
Flavonoids	LMPK12130058	Ambofuracin	571.180	7.870	36	Positive	2.050	0.639	0.036
	LMPK12110281	Vitexin 3''',4'''-Di-O-acetyl 2''-O-rhamnoside	661.176	7.872	35.6	Negative	2.340	0.310	0.016
	LMPK12080046	7-O-Methyllicoricidin	921.481	8.193	38.6	Negative	3.543	2.072	0.000
Others	HMDB06117	APGPR Enterostatin	519.264	8.301	37.3	Positive	2.346	0.265	0.017
	HMDB07065	DG(14:1(9Z)/24:1(15Z)/0:0)	671.559	10.020	36.4	Positive	2.108	0.547	0.030
	LMST05010016	6alpha-Glucuronosylhyodeoxycholate	284.666	6.060	37.8	Positive	1.989	0.650	0.042
	LMST03020073	1alpha-hydroxy-24-(dimethoxyphosphoryl)-25,26,27-trinorvitamin D3	484.320	5.307	37.2	Positive	2.555	2.652	0.012
	LMGP06010417	PI(18:4(6Z,9Z,12Z,15Z)/20:5(5Z,8Z,11Z,14Z,17Z))	899.469	8.198	35.3	Positive	3.902	2.549	0.000
	LMPK12140228	Flavaprenin 7,4'-diglucoside	665.246	3.000	36.6	Positive	2.214	0.412	0.023
	LMPK04000042	Troleandomycin	831.483	8.191	35.8	Positive	3.751	1.828	0.000
	LMST04010192	3alpha-Hydroxy-7,12-dioxo-5beta-cholan-24-oic Acid	449.255	5.358	39	Negative	3.282	0.317	0.000
	HMDB00226	Orotic acid	357.032	0.822	38.3	Negative	1.945	0.665	0.047
	LMGP03050014	PS(22:4(7Z,10Z,13Z,16Z)/0:0)	594.282	5.940	36.9	Negative	2.108	0.562	0.030

Compound ID was mainly exhibited based on the Human Metabolome Database (www.hmdb.ca) and LIPID MAPS (www.lipidmaps.org); FC value was calculated as the ratio of the average mass response (area) between the two groups (FC value = Females/males)

*P* < 0.05 indicates significantly differences between the two groups. RT, retention time; MS, Matching score; VIP, variable influence on projection; FC, fold change

**Fig. 4:**
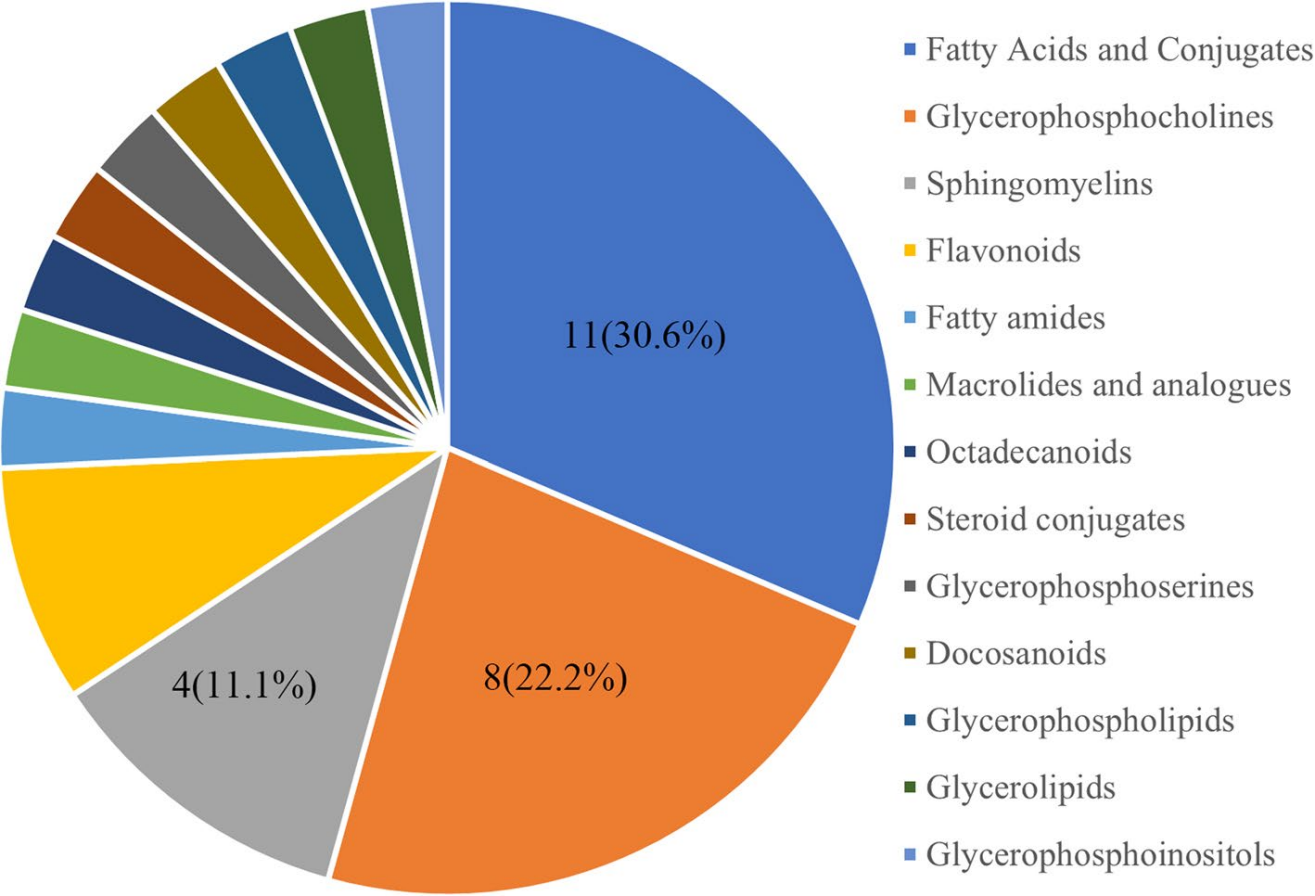
Pie chart depicting the classification of differentially expressed metabolites

The differentially expressed metabolites can be categorized into fatty acids and conjugates, glycerophosphocholines (GPCs), sphingomyelins, macrolides and analogues, steroid conjugates, flavonoids, fatty amides, octadecanoids, docosanoids, glycerophosphoserines, glycerophosphoinositols, glycerophospholipids, glycerolipids, and so on. The top four main classes of metabolites were fatty acids and conjugates (11, 30.6%), GPCs (8, 22.2%), sphingomyelins (4, 11.1%), and flavonoids (3, 8.3%) (Fig. 4). According to the metabolite sets enrichment analysis, fatty acids and conjugates, GPCs, and sphingomyelins were significantly enriched in the over-representation analysis (Table 3).

**Table 3:** Metabolite sets enrichment analysis of the differentially expressed metabolites between males and females of all participants

Main class	Total compounds	Hits	*p* value	FDR
Fatty Acids and Conjugates	3090	11	2.53E-07	6.17E-05
Glycerophosphocholines	4700	8	4.12E-06	0.000503
Sphingomyelins	2320	4	0.000347	0.0283
Macrolides and analogues	8	1	0.00117	0.0713
Steroid conjugates	123	1	0.0178	0.87
Flavonoids	5300	3	0.0415	1
Fatty amides	406	1	0.0577	1
Octadecanoids	498	1	0.0703	1
Docosanoids	740	1	0.103	1
Glycerophosphoserines	4140	1	0.458	1
Glycerophosphoinositols	4360	1	0.475	1
Glycerophospholipids	36,400	1	0.997	1
Glycerolipids	41,400	1	0.999	1

*FDR* false discovery rate

## Discussion

Lipid metabolic disturbances are found in various diseases, including metabolic syndrome, cardiovascular disease, heart failure, cerebrovascular disease, and Guillain–Barre syndrome [13]. Lipid metabolism is correlated with Parkinson’s disease and related neuropsychiatric symptoms according to our former research [14]. The occurrence and development of diabetic peripheral neuropathy can be delayed by regulating lipid metabolism [15]. Sex differences were also found in very-low-density lipoproteins triglyceride and low-density lipoprotein cholesterol with age dependence [1]. We were the first to perform LC–MS-based metabolomics in Chinese aged population to clarify sex differences and found some specific lipid changes as follows.

### Fatty acids and conjugates

The group of fatty acids and conjugates is a subclass of fatty acyls, which is among the eight categories of lipids in current classification system. It is associated with the occurrence and development of many diseases. The plasma levels of long-chain omega-3 and long-chain omega-6 fatty acids were associated with a lower risk of schizophrenia while short-chain omega-3 and short-chain omega-6 fatty acids were associated with an increased risk of schizophrenia [16]. The supplementation of omega-3 fatty acid can reduce major adverse cardiovascular events, cardiovascular death, and myocardial infarction [17]. It is reported that in tambaqui (Colossoma macropomum) elongation of very long-chain fatty acids enzymes and fatty acid metabolism played important roles in sexual differentiation [18]. A former gas-chromatography mass-spectrometry-based metabolomic analysis with urine samples from healthy males and females indicated that saturated fatty acids were significantly correlated with sex [19]. In this research, eleven differentially expressed fatty acids and conjugates were found and nine were significantly increased in males. These nine metabolites varied in molecular structures without obvious characteristics, including dicarboxylic acid, aromatic compound, monounsaturated fatty acid, polyunsaturated fatty acid, and the carbon numbers of those molecules ranged from eight to twenty-three. The sex-specific expression mode of fatty acids and conjugates may contribute to the differential incidences of schizophrenia and cerebral vascular diseases in males and females.

### Glycerophosphocholines

GPCs are glycerophospholipids in which a phosphorylcholine moiety occupies a glycerol substitution site. GPCs are mainly exogenous and abundant in egg, soybean, beef, shrimp, and any other foods. The preferences and consumption structures of food between the two sexes lead to the different content levels of GPCs. GPCs can cross the blood–brain barrier and serve as contributors of choline and phospholipid in central nervous system. GPCs are reportedly involved in depression, anxiety, dementia, and many other neurological disorders. They can be hydrolyzed by the enzyme phospholipase A2 into lysophosphatidylcholines (LysoPCs) while lysoPCs can specifically bind to the G protein-coupled receptor family (GPR119, GPR40, GPR55 and GPR4), and induce intracellular calcium mobilization leading to increased glucose-stimulated insulin secretion. LysoPCs also have several protective or anti-inflammatory effects and serve as dual-activity ligand molecules in the innate immune system [20]. A total of eight GPCs had been found differentially expressed between males and females. The contents of LysoPCs were significantly higher in males while the lipid chains of males were significantly longer than females.

### Sphingomyelins

Sphingomyelins help form lipid rafts in cell membranes and are involved in signal transduction and transportation of lipids and proteins [21]. It can be hydrolyzed by sphingomyelinases into ceramides, which are important second messengers in cell proliferation, differentiation, proliferation, and apoptosis [22]. The balance of sphingomyelins is essential for normal neuronal function and the deficiencies in enzymes of sphingomyelins metabolism can lead to various severe brain disorders. Some researches indicated blood-based sphingomyelins played crucial roles in dementia but the conclusions were inconsistent [23]. A longitudinal cohort provided evidence for sex-specific associations between sphingomyelins and dementia, which might account for the above inconsistence [24]. The changes of plasma sphingomyelins with age were statistically different by sex, and sphingomyelins decreased in males but increased in females with age [25]. Sex differences of sphingomyelins were also found in the development of stress-induced depression [26].

There were several limitations to this study. Firstly, the sample number of included participants was relatively small and further confirmation is needed. Secondly, more experimental methods should be performed to extensively identify sex differences of metabolism in plasma, including gas-chromatography mass-spectrometry and nuclear magnetic resonance profiling.

## Conclusions

Significant lipid metabolic differences were found between the two sexes among the elderly. Fatty acids and conjugates, glycerophosphocholines, and sphingomyelins may partly account for sex differences and can be potential treatment targets for sex-specific diseases.

## Data Availability

The datasets used and/or analysed during the current study are available from the corresponding author (Mei-Xue Dong, Email:dong_meixue@whu.edu.cn) on reasonable request.

## Abbreviations

UPLC: Ultra Performance Liquid Chromatography
PCA: Principal component analysis
OPLS-DA: The orthogonal partial least squares-discriminant analysis
GPCs: Glycerophosphocholines
LysoPCs: Lysophosphatidylcholines
